# Effect of *Boswellia* species on the metabolic syndrome: A review

**DOI:** 10.22038/ijbms.2020.42115.9957

**Published:** 2020-11

**Authors:** Davood Mahdian, Kazem Abbaszadeh-Goudarzi, Amir Raoofi, Ghazaleh Dadashizadeh, Mina Abroudi, Elahe Zarepour, Hossein Hosseinzadeh

**Affiliations:** 1 Cellular and Molecular Research Center, Sabzevar University of Medical Sciences, Sabzevar, Iran; 2 Department of Pharmacology, School of Medicine, Sabzevar University of Medical Sciences, Sabzevar, Iran; 3 Pharmaceutical Research Center, Pharmaceutical Technology Institute, Mashhad University of Medical Sciences, Mashhad, Iran; 4 Department of Pharmacodynamics and Toxicology, School of Pharmacy, Mashhad University of Medical Sciences, Mashhad, Iran

**Keywords:** Boswellia, Dyslipidemia, Frankincense, Hyperglycemia, Hypertension, Metabolic syndrome, Olibanum

## Abstract

The metabolic syndrome, a cluster of metabolic disorders, includes abdominal obesity, hypertension, dyslipidemia, and hyperglycemia leading to insulin resistance, development of diabetes mellitus, and cardiovascular diseases. For the treatment of metabolic syndrome, traditional herbal medicines such as frankincense or *Boswellia* species have been used due to their anti-inflammatory, anti-oxidant, anti-obesity, antidiabetic, antihypertensive, and hypolipidemic properties. Based on the literature, published evidence up to 2020 about the therapeutic effects of *Boswellia* species on the metabolic disorder among Medline, Scopus, and Google Scholar were precisely evaluated by keywords such as obesity, diabetes, hyperglycemia, hypertension, blood pressure, dyslipidemia, metabolic syndrome, frankincense, and Boswellia. According to the results, *Boswellia* species have beneficial effects to control metabolic syndrome and its related disorders such as hyperglycemia, dyslipidemia, hypertension, obesity, diabetes, and its complications. *Boswellia* species by reducing the resistance to insulin and restoring pancreatic beta cells decrease blood glucose. Also, *Boswellia* species has antithrombotic and anticoagulant properties that regulate blood pressure. The anti-oxidant properties of *Boswellia* species modulate the blood lipid profile via reducing TNF-α, IL-1β levels, and increasing the adiponectin level. The therapeutic and protective effects of *Boswellia* species on metabolic disorders were remarkably confirmed regarding decreasing hyperglycemia, hyperlipidemia, hypertension, and obesity.

## Introduction

The metabolic syndrome is defined as abdominal obesity, dyslipidemia (DL), hypertension (HTN), insulin resistance with or without glucose tolerance, pro-inflammatory and pro-thrombotic state (1, 2). Moreover, metabolic syndrome is a serious problem and challenge with an ascending trend throughout the world that is caused by excessive excess calorie intake, urbanization, and inactive lifestyles (3). Systemic HTN that is also known as high blood pressure (HBP) and abnormal lipid profile can be observed because of the resistance to insulin, which results in atherosclerotic vascular disease. Hence, metabolic syndrome enhances myocardial infarction (MI) and stroke risks (4).

Today, studies have been focused on supplementary and substitute medicine (5-7) due to inadequate efficiency and significant complications from recent treatments for hyperlipidemia (HLP) and diabetes (8-11). Recently, researchers have paid attention to the use of medicinal plants due to the reduced complications and different efficient compounds in the herbs as recommended by the World Health Organization (WHO) (12).

Concerning the important clinical consequences of the metabolic syndrome, investigators have mainly attended to study the values of herbal medicines or herbalism. *Boswellia* internationally known as Indian frankincense or olibanum has been used to treat various diseases. The frankincense or olibanum, a yellowish-brown oleo-gum resin, is prepared from *Boswellia* species such as *Boswellia serrata* (13). *Boswellia* genus contains about 25 different species. A number of the prominent species consist of *B. frereana, B. sacra, B. ovalifoliolata, B. carterii, B. papyrifera, B. rivae, B. neglecta*, and so forth (14-17). Recent research indicated the anti-inflammatory, anti-ulcerous function, and anticancerous impacts of this plant (18). Of course, some studies demonstrated the antihyperglycemic and antihyperlipidemic impacts of *Boswellia* in streptozotocin-induced diabetic rats (19). Also, one of the studies indicated the protective impacts of *B.* *serrata* gum on diabetic side effects in animal models (20, 21). Moreover, this plant has useful impacts on the low-density lipoprotein (LDL), blood glucose, and high-density lipoprotein (HDL) of patients with diabetes who had received *B.* *serrata* gum at a dosage of 900 mg every day with no significant complications (22). 

Numerous animal research projects demonstrated the anti-oxidant features of gum resin extracts of *B.* *serrata* (23, 24). Pandey *et al.* showed that the extracts of *B.* *serrata* gum resin resulted in a reduction in serum cholesterol and the enhancement of HDL in the rats (25). Another research indicated that patients with type 2 diabetes who have been supplemented with gum resin of *B.* *serrata* for six weeks experienced a remarkable decline of fasting blood glucose and the augmentation in plasma insulin level (26).

**Pharmacognostical features of** ***Boswellia***

*Boswellia* belongs to the family Burseraceae, which is a deciduous tree. In general, it reaches a moderate height (4 to 5 m). As other moderate to big size trees with branches, this tree possesses a circumference of 2.4 m (average 1.5 m). The color of the thin barks of the tree changes from greenish-gray, yellow, or reddish to ash color that its peeling may be readily done. The papery barks, when peeled off or cut, release translucent lumps, tear, or droplets of white to yellow color gummy oleoresin (26). 

**Composition**

There are nearly 200 phytochemicals in the oleo-gum-resin mix in various species of *Boswellia*. These compounds contain pure resin, mucus, and essential oil (27, 28). 

**Figure 1:**
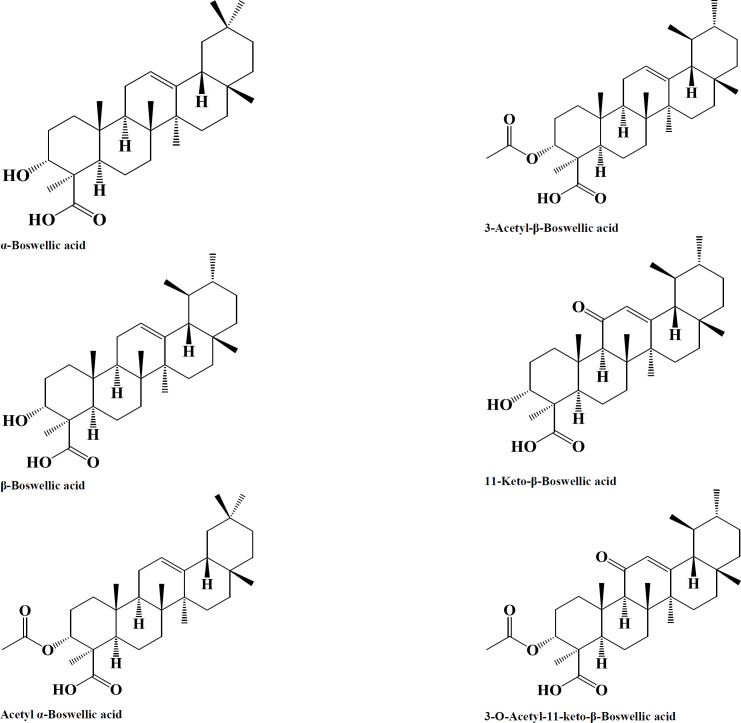
Boswellic acid and its derivatives

The essential oil compositions of each species are different and change concerning the environment, harvest condition, and geographic areas (29, 30). The gum portion is composed of pentose and hexose sugar containing a couple of oxidation and digestive enzymes. The essential oil is a mix of mono-terpenes, diterpenes, and sesquiterpenes. β-boswellic acid (Bas) is the main constituent of each species of the genus *Boswellia* (Figure1). There are 6 main Bas, including α- and β-BA (10 to 21%), acetylated α- and β-BA (0.05–6%), 11-keto-β-boswellic acid (KBA, 2.5 to 7.5%), and 3-O-acetyl-11-keto-β-boswellic acid (AKBA, 0.1– 3%), which are found in each *Boswellia* species with variable amounts. The contents of BA, which can be found in the market as the standardized extracts, change from 37.5-65% (31, 32).

**Review literature**

The search was performed by keywords such as diabetes or hyperglycemia, *Boswellia*, frankincense, olibanum, boswellic acid, elevated BP or HTN, hypotensive or antihypertensive, DL, and metabolic syndrome using Google Scholar, Scopus, and Medline databases. In fact, in the current research, most of the papers about the effects of *Boswellia* on metabolic disorders have been precisely evaluated. To better review, irrelevant or duplicated papers have been disregarded. Publications have been specified from their admission up to August 2020.

**Dosing**

*Boswellia* species are usually administered as a capsule, pill, or bark decoction orally. The respective dose is suggested based on historical practices or existing trials. Today, there is ambiguity on the optimum dosage for balancing a safe and efficient method. Each producer has their production method of *Boswellia*, and these results are too inconsistent to provide a standardized product. Notably, several trials applied different products manufactured by different producers. For this reason, clinical impacts cannot be compared (33).

**Hypoglycemic effect of the** ***Boswellia species*** **in diabetic patients and animal models**

*Boswellia* species tree and the corresponding gummy resin are completely known due to their useful impacts on several diseases such as diabetes mellitus (DM) (39). Azemi *et al.* showed that the extract of *B.* *serrata* has antidiabetic impacts and can prevent the microvascular complications of diabetes in the kidney and liver (40). Investigators showed that herbal formulations with *B.* *serrata* oleo-gum-resin generated considerable antidiabetic activities via influencing the hepatic gluconeogenesis, pyruvate carboxylase, and phosphoenolpyruvate carboxykinase (41). Shehata *et al.* studied AKBA contribution to the prevention of inducing auto-immune reaction, insulitis, and hyperglycemia in a model of multiple low dose streptozotocin (MLD-STZ) diabetes. The induction of hyperglycemia or high blood sugar has been done via injection of IP 40 mg/kg STZ in male mice for five days every day, whereas the second treatment group has been administered KBA with STZ for ten days. In STZ treated rats, a considerable burst of pro-inflammatory and anti-inflammatory cytokines in the blood, infiltrating lymphocytes (CD3) into pancreatic islets, and emerging peri-insular apoptotic cells have been registered. A significant increase in plasma glucose has been observed (124.4±6.65 versus 240.2±27.36 mg/dl, *P*<0.05). Nonetheless, concurrent treatments with AKBA and KBA indicated a significant decrease in pro-inflammatory and anti-inflammatory cytokines. Moreover, the detection of infiltrating lymphocytes into pancreatic islets and emerging peri-insular cells has not been reported (42).

Ahangarpour *et al*., clinically studied patients with type 2 diabetes supplemented with extract of *B.* *serrata* gum resin for six weeks and compared them to type 2 diabetic patients. A considerable reduction of fasting blood glucose and a decrease in insulin levels have been reported (22). The researchers were satisfied with the findings, and thus developed the research and examined antidiabetic, hypolipidemic, and hepatoprotective impacts of the supplement *B.* *serrata* in 60 patients with type 2 diabetes from both genders. Treating diabetic patients with the extract of *B.* *serrata* gum resin (orally, 900 mg) for six weeks led to a considerable enhancement of the levels of HDL and significant decline of cholesterol, LDL, as well as the amounts of fructosamine SGPT and SGOT. The research indicated that the administration of 900 mg of *B.* *serrata* supplement daily is one of the healthy and efficient options for decreasing hazardous agents related to type 2 diabetes. Diabetic patients who receive *B.* *serrata* can keep fructosamine level, hepatic enzyme activity, and lipid profiles close to the standard levels and have a high-quality life (21). Herbal formulations with *B.* *serrata* oleo-gum-resin as an ingredient of the supplementation led to considerable antidiabetic activities on non-insulin-dependent DM in streptozocin induced diabetic rat model.

In a case study report of a male patient diagnosed with Latent Autoimmune Diabetes in Adults, *B.* *serrata* gum resin during 9 months treatment reduced both the markers of autoimmune diabetes, i.e., GAD65 and IA2 autoantibodies (43).

Some results demonstrated the decline of the blood glucose levels in patients with diabetes by orally administrating the aqueous extracts of the leaf and root of *B. glabra*. The continuous application of the extract of the leaf and root for 28 days represented a reduction of cholesterol, creatinine, triglyceride, serum glucose, urea, enzyme activity with considerable hypo-glycemic impacts (44). One of the auto-immune diseases is a type I diabetes, in which a chronic inflammatory procedure eventually leads to beta-cell mortality and a lack of insulin production. It was indicated that extracts obtained by the gum resin of BS have anti-inflammatory features, especially via targeting agents or mediators associated with auto-immune diseases (39). The recent research demonstrated the antidiabetic impacts of BS extracts and their capability for preventing the side effects of diabetes in the kidney and liver (40).

Hypoglycemic activities in mice with type 1 diabetes have been confirmed by BS oleo-gum and respective active ingredients, KBA and AKBA by suppressing pro-inflammatory cytokines related to inducing auto-immune procedure in pancreatic islets, such as interleukin (IL)-1A, IL-1B, IL-2, IL-6, interferon (IFN)-γ, TNF-α, granulocyte colony-stimulating factor (G-CSF), and granulocyte/macrophage colony-stimulating factor (GM-CSF), and infiltrating lymphocytes into islets. Two of the major antidiabetic mechanisms include suppressing pancreatic islet tissue atrophy and peri-insular apoptotic cells mediated by anticaspase 3 (39, 45). Rao *et al.* (2013) revealed the improvement of chronic diabetic side effects by oleo-gum resin and the isolated compound boswellic acid through the inhibition of polyol enzyme aldose reductase and reduction in the developed glycation end product *in vivo* in rat lens and rat kidneys and *in vitro* in human recombinant cells (20). Additionally, *B. carterii* oleo-gum resin showed an antidiabetic capacity by increasing the serum insulin, regenerating β-cells of Langerhans islets, enhancing glycogenesis, and declining glycogenolysis in rats with alloxan-induced type 1 diabetes (46).

The previous study showed that blood-glucose levels increased significantly (*P*<0.05) in the control group in comparison to other groups that received 3 g *B.* *serrata*/l in drinking water. However, the remaining treated groups showed a significant decrease in comparison with control (47). Also, Al-Daraji *et al.* (47), reported that the drinking water of broiler chickens supplemented with different levels of *B. carterii *powder led to a significant decrease in blood glucose concentrations, at levels 0.5, 0.75, and 1 g/l. However, 0.75 and 1 g/l water supplementation reduced the values of blood plasma concentrations of glucose. 6 weeks complementarity of *B.* *serrata* to type 2 diabetic patients also produced a very significant decrease in fasting blood glucose and an increase in insulin level (48). Similarly, *B. glabra* aqueous extract increased the synthesis of secretory granules in the beta-cell and led to an increase in pancreatic enzyme resulting in reduced blood-sugar level (49) (Tabel 1).

**Impact on the elevated blood pressure**

Not many authors assessed the positive impacts of *Boswellia* species on elevated BP which is an important component of the metabolic syndrome. Recent studies have introduced some mechanisms of actions concerning *B.* *serrata* gum resin on cardiac health. There is enough knowledge of the relationship between oxidative stresses, inflammation, and thrombosis resulting in cardiovascular diseases (50, 51). Hence, the anti-oxidant and antithrombotic characteristics of *B.* *serrata* gum resin were examined. Enriching *B.* *serrata* gum resin with triterpenoids showed their anti-oxidant activities based on the respective chemical compositions (52, 54). The experiments of the anti-oxidant activities of *B.* *serrata* gum resin suggested antilipid per-oxidation actions in the liver and heart. Researchers studied the phytochemical ingredients of the crude extract of *B.* *serrata* and demonstrated that it consists of essential oils, resin, and gum. Boswellic acid, a pentacyclic triterpene, has been recognized as an active moiety of the resin portion (55). Primary phytochemical studies showed the existence of flavonoid and saponin in *B.* *serrata* Previous observations showed that several compounds, such as flavonoids, saponins, or organic acids might contribute to the herb diuretic impacts (56). Likewise, some authors revealed that specific flavonoids induce diuretic activities by attaching with adenosine A1 receptors related to the diuretic actions (57). Since *B.* *serrata* has a lot of saponins and flavonoids, the diuretic activities of the herb understudy might result from these mechanisms. It has been indicated that sodium is a prominent external agent that plays a role in primary HTN (58). Several research projects demonstrated the adverse effects of higher uptake of sodium adversely on the arterial BP (59). The higher excretion of urinary sodium in the present experimental animals revealed the antihypertensive activity of the *B.* *serrata* (Table 1).

**Impact on obesity and lipid profiles**

Several herbal medicines such as *Ginkgo biloba *can manage and improve hyperlipidemia or obesity in patients (60). Numerous academic research projects performed during recent years showed that *Boswellia* species would be efficient hypolipidemic agents. It has been demonstrated that the water-soluble fraction of *B.* *serrata* reduces the levels of total cholesterol (38-48%) (61) in experimental animals, which confirms its hypolipidemic potentials. Moreover, Zutshi *et al.* showed the antihyperlipidemic activities of *Boswellia* gum (19). Salami gum keeps the levels of serum cholesterol and triglyceride of animals within optimal ranges that would be received on diets with increased cholesterol and saturated fat (61). It was reported that AKBA inhibits NF-κB activity in atherosclerosis (62). Of course, AKBA has anti-adiposity properties, through which it can induce lipolysis in mature human adipocytes that have been observed by Liu *et al.* in the* in vitro* study. Moreover, this event has been followed by downregulating the expression of PPAR-g2 and losing phenotypic markers (63). The study of Al-Yasiry *et al.* (46) showed that *Boswellia* species (3 g *B.* *serrata*/l in drinking water) reduced the cholesterol level in broiler chicken. Their findings were in agreement with those of Pandey *et al* (64). In this study, they showed that the supplementation of BS gum resins extract 15 mg/100 g body wt for 90 days caused a significant decrease in serum cholesterol and increased HDL in rats. It has also been reported by Al-Daraji *et al.* (65) that *B. carterii* (0.5, 0.75, and 1 g/l in drinking water) decreased significantly cholesterol, triglycerides, and LDL levels. The results of this study suggest the probability that *B.* *serrata* supplementation restores β-cells function for insulin secretion, and that insulin helps to reduce serum lipid profiles (66). Moreover, *B. carterii* may have a protective effect on pancreatic β cells through its anti-oxidant action (67) (Table1). Obesity, a chronic disease characterized by the storage of excess energy in fat cells, is a result of abnormal metabolism. Obesity is a complex issue and its causes, consequences, and management are an area of considerable debate as a widespread disease. Several studies reported *Boswellia* species exhibited anti-obesity effects by lowering total cholesterols, triglycerides, free fatty acids, LDL concentrations, circulating adiponectin, food intake as well as elevating HDL (68-70). The study performed by Tawfik showed that boswellic acid has a promising anti-aggregatory effect by reducing the enhanced HLP, oxidative stress, and inflammation associated with a high-fat diet (HFD) (71). Gomaa *et al.* investigated that *B.* *serrata* extract is as effective as orlistat in preventing obesity, hyperlipidemia, steatosis, and insulin resistance. These actions may be mediated by the suppression of food intake and reducing the levels of TNF-α, IL-1β, and leptin resistance along with increasing adiponectin (72). *B.* *serrata* extract has anti-obesity effects and can be attributed to the presence of active principles such as phenolic compounds and triterpenoids (73-75). In another study, *B.* *serrata* extract showed a suppressive effect on cumulative food intake compared to ephedrine used as a standard anorectic drug (76). 

The findings of previous studies demonstrated that the use of *B.* *serrata* appears safe and effective to control obesity (77). The possible mechanism of *B.* *serrata* reported by Singh *et al.* consists of the stimulation of the thyroid gland leading to an increase in metabolic rate. Thereby enhancing thyroid efficiency which in turn causes to lose weight. Regarding toxicity, *B.* *serrata* showed no toxic effect up to 500 mg/kg (78) (Figure1 and Table1).

**The overall mechanism of** ***Boswellia species*** **in the metabolic syndrome**

Several features of *Boswellia* species have been explored. The general mechanisms of the *Boswellia* species include anti-oxidant, radical scavenger, glutathione contents regulator, cellular membranes stabilizer, and cell permeability regulator. Moreover, *B.* *serrata* extracts enhance the regeneration of the liver and delay developing and progressing hepatic fibrosis (34, 35).

**Figure 2:**
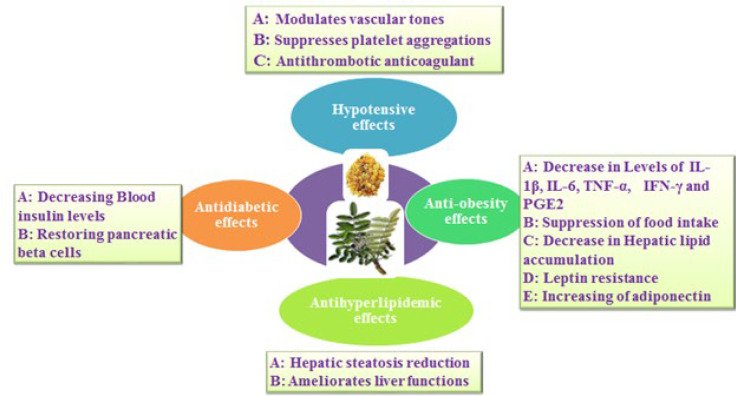
Schematic description of the traditional effects of *Boswellia* on the metabolic syndrome

Based on numerous research projects, the major classifications of *Boswellia* mechanisms are as following:

1. *Boswellia* gum resin reduces plasma glucose in diabetes by decreasing the resistance to insulin and restoring pancreatic beta cells (36) (Figure2). 

2. *Boswellia* gum resin regulates BP in hypertensive conditions by modulation vascular tones, diuretic effects, and suppression platelet aggregations with antithrombotic and anticoagulant properties (37) (Figure2). 

3. *Boswellia* gum resin adjusts the lipid profile via decreasing hepatic steatosis and ameliorates liver dysfunctions tests through its anti-oxidant and cytoprotective impacts (38) (Figure2). 

4. *Boswellia* extracts suppress food intake and reduce TNF-α, IL-1β levels and leptin resistance along with increasing the adiponectin level (72) (Figure2).

**Table 1:** The efficacy of *Boswellia* species on different animal models composed of the metabolic syndrome

Study type	Metabolic syndrome component	Reference
Animal studies	↓ Blood insulin levels	
Animal studies	↓ Hyper-glycemia	
Human studies	↓ Fasting blood glucose ↓ Insulin levels	
Animal studies	↓Hepatic gluconeogenesis, ↓Blood glucose	
Human studies	↓ Diabetic Hypolipidemic ↑ Levels of HDL ↓ Total cholesterol, triglycerides, LDL	21,65,66
Animal studies	↓ Blood glucose ↓ Cholesterol, creatinine, triglyceride, serum glucose, urea, enzyme activity	44,49,50,61,62,46
Animal studies	↓ Diabetic ↓ Pancreatic damages ↓ Infiltration of lymphocytes into pancreatic islets	39,45
Animal studies	↓ Polyol enzyme aldose reductase ↓ The developed glycation ↓ Blood glucose	
Animal studies	↓ Blood insulin levels	
Animal studies	↓ Blood insulin levels	
Animal studies	↓ The serum insulin ↑ Glycogenesis ↓ Glycogenolysis	
Animal studies	Adjusts the lipid profile	
Animal studies	↑ Anti-oxidant defense ↑ Anti-oxidant and Anti thrombotic effects ↑ Antilipid peroxidation actions in liver and heart ↑Diuretic activities ↑Antihypertensive factor ↓ High sodium absorption	53,55,57,58
Animal studies	Modulates vascular tones	
Animal studies	↓ Obesity ↓Food intake ↓Concentration adiponectin ↓Hyperlipidemia ↓Oxidative stress and inflammation ↓TNF-α, IL-1β ↓leptin resistance	72,75,76
Animal studies	↓Oxidative stress and inflammation ↓TNF-α, IL-1β ↓Leptin resistance	75,73
Human studies	↓ Obesity	

DM: Diabetes Mellitus; NAFLD: Non-Alcoholic Fatty Liver Disease; HLP: Hyperlipidemia; LDL: Low-Density Lipoprotein; HDL: High-Density Lipoprotein; BP: Blood Pressure, OB: Obesity

## Conclusion

According to the broad range of properties of *Boswellia* species, this review described the potential effects of *Boswellia* species in either the treatment or prevention of the metabolic syndrome. The previous studies shed light on new ways of treatment for the metabolic syndrome by the exhibition of the effectiveness of *Boswellia* species in HBP, obesity, DL, and high blood glucose. Nevertheless, a series of effective clinical studies should be conducted in this regard. 

## Conflicts of Interest

The authors declare that they have no known competing financial interests or personal relationships that could have appeared to influence the work reported in this paper. 
